# Beyond the Bench: Promoting Health in Texas *Colonias*

**Published:** 2005-07

**Authors:** Tanya Tillett

Sometimes the best educational resources can be found in your own backyard. Staff at the Community Outreach and Education Program (COEP) of the NIEHS Center for Environmental and Rural Health at Texas A&M University recognize how true that can be. Concerned with the health of residents in local *colonias* (poor, unincorporated neighborhoods along the Texas–Mexico border whose residents are largely of Mexican origin), COEP staff have created a dynamic program that trains *promotoras*—residents of the *colonias*—to serve as a link between communities and health educators.

The effort began six years ago as a way to educate *colonia* residents near Laredo about pesticides and other hazards. These residents lived near farm fields, and pesticides were showing up in house dust and hand-rinse samples. The program now includes a comprehensive environmental health educational program reaching *colonia* residents in areas bordering Laredo, McAllen, and Bryan.

The *promotoras* are crucial to the success of the *colonia* outreach program because they are better able to establish a dialogue with community members. Families then often feel freer to express their concerns with regards to environmental health and more receptive to health education materials. The *promotoras* usually live in the same neighborhood they provide outreach to, lending a sense of trust and familiarity to their interactions with other residents.

*Promotoras* are recruited through the Center for Housing and Urban Development at Texas A&M University and through the South Texas Association of *Promotoras*. According to COEP director Carmen Sumaya, it takes a special person to be a *promotora*. Although they vary in age from 25 to 60, with consequent variations in life experience, all *promotoras* are natural community advocates. These volunteers are willing to devote the time to absorb environmental health information and make it relevant to their neighbors so they can take something useful back to their communities.

Melly Tamez, a Laredo *promotora*, is a dedicated community advocate who sees a need for such information in her community and works to provide it. “The people in my neighborhood need help in health issues, and I want to improve the quality of their lives by instructing them on how to avoid environmental health risks. I take great pride in my role as *promotora*,” says Tamez.

The first phase of the program involves COEP staff and the *promotoras* meeting with families at local community centers so residents can identify any environmental health concerns they have. The second phase involves training the *promotoras* to use flip charts and other materials to convey relevant environmental health information (for example, safe drinking water and food safety practices). The *promotoras*’ feedback is important at this stage because they can help clarify how health messages can be presented most effectively. In the third phase, the *promotoras* schedule and conduct visits with their neighbors to provide culturally relevant environmental health education.

In a typical visit, two *promotoras* come to the home of a resident who has invited at least two neighbors to participate. While one *promotora* presents environmental health information to the adults, the other engages the children of the household with coloring books and other fun activities. After the presentation, the *promotoras* answer any questions the residents might have and schedule follow-up visits for one and three months later.

To date, about 15 *promotoras* have been trained by the COEP. Sumaya acknowledges their value to the COEP’s environmental health campaign. “*Promotoras* open the door for us to the people of the *colonias*. They play a pivotal role in the success of our outreach programs,” she says.

## Figures and Tables

**Figure:**
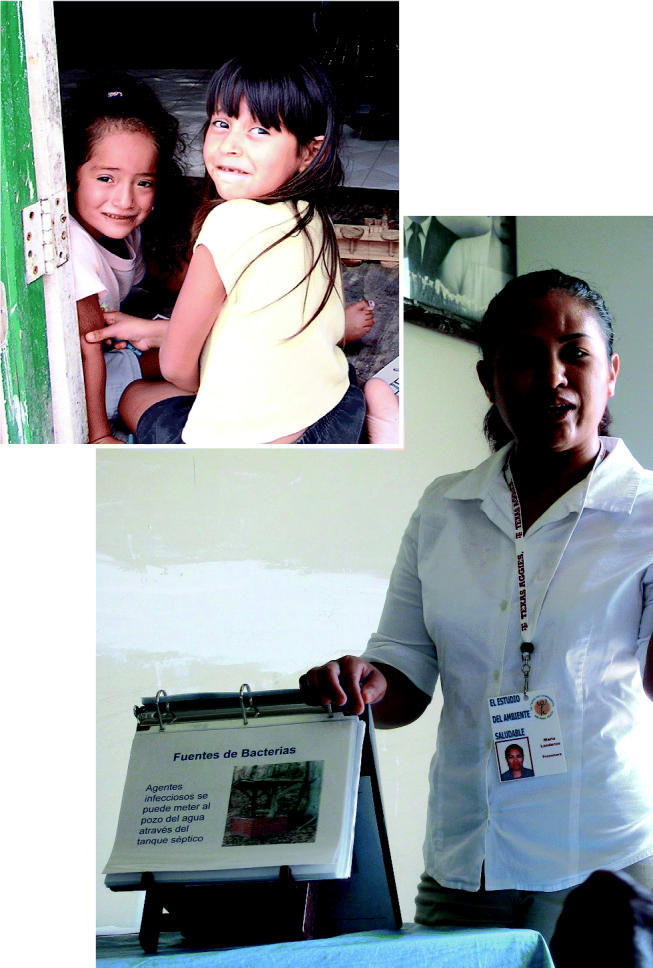
**Neighborly advice.** A program along the Texas–Mexico border trains community members to be health advocates.

